# Supplementary material for: Covalently-assembled single-chain protein nanostructures with ultra-high stability
Source: Nat Commun. 2019 Jul 25;10:3317. doi: 10.1038/s41467-019-11285-8 (PMC6658521; doi:10.1038/s41467-019-11285-8)
Supplement: Supplementary file 1 — Supplementary Information [file 41467_2019_11285_MOESM1_ESM.pdf]

Supplementary Information

Title: Covalently-assembled single-chain protein nanostructures with ultra-high stability

Authors: Wenqin Bai[1], Cameron J. Sargent[2], Jeong-Mo Choi[3], Rohit V. Pappu[3], Fuzhong Zhang[1, 2, 4*]

Affiliations:

[1]Department of Energy, Environmental and Chemical Engineering,

[2] Division of Biological & Biomedical Sciences,

[3] Department of Biomedical Engineering and Center for the Science & Engineering of Living Systems,

[4] Institute of Materials Science & Engineering,

Washington University in St. Louis, Saint Louis, MO 63130, USA

[*]Correspondence to: fzhang@seas.wustl.edu

Supplementary Figures

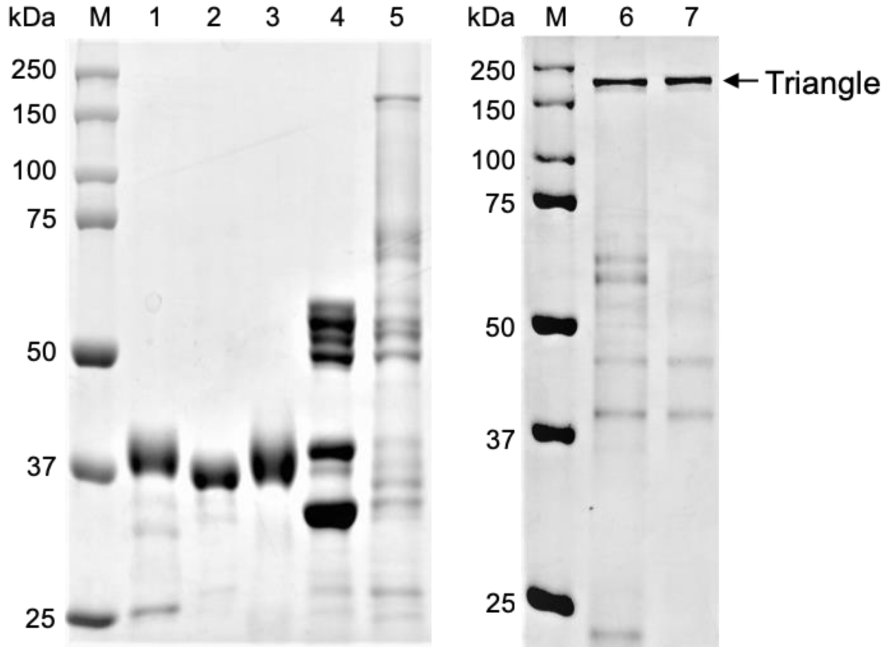

Supplementary Figure 1 SDS-PAGE gels of the construction and purification of triangular nanostructure Tri18. Lane M, protein marker; lane 1, purified Gp^{C}_{18}-3HB-Cfa^{N}_{14}; lane 2, purified Cfa^{C}_{14}-3HB-$NrdJ^{N}_{18}$; lane 3, purified $NrdJ^{C}_{18}$-3HB-Gp^{N}_{18}; lane 4, ligation product of Cfa^{C}_{14}-3HB-$NrdJ^{N}_{18}$ and $NrdJ^{C}_{18}$-3HB-Gp^{N}_{18}; lane 5, ligation product of Cfa^{C}_{14}-3HB-$NrdJ^{N}_{18}$, $NrdJ^{C}_{18}$-3HB-Gp^{N}_{18}, and Gp^{C}_{18}-3HB-Cfa^{N}_{14}; lane 6, triangle purified by Ni-NTA column; lane 7, triangle further purified by SEC. Source data are provided as a Source Data file.

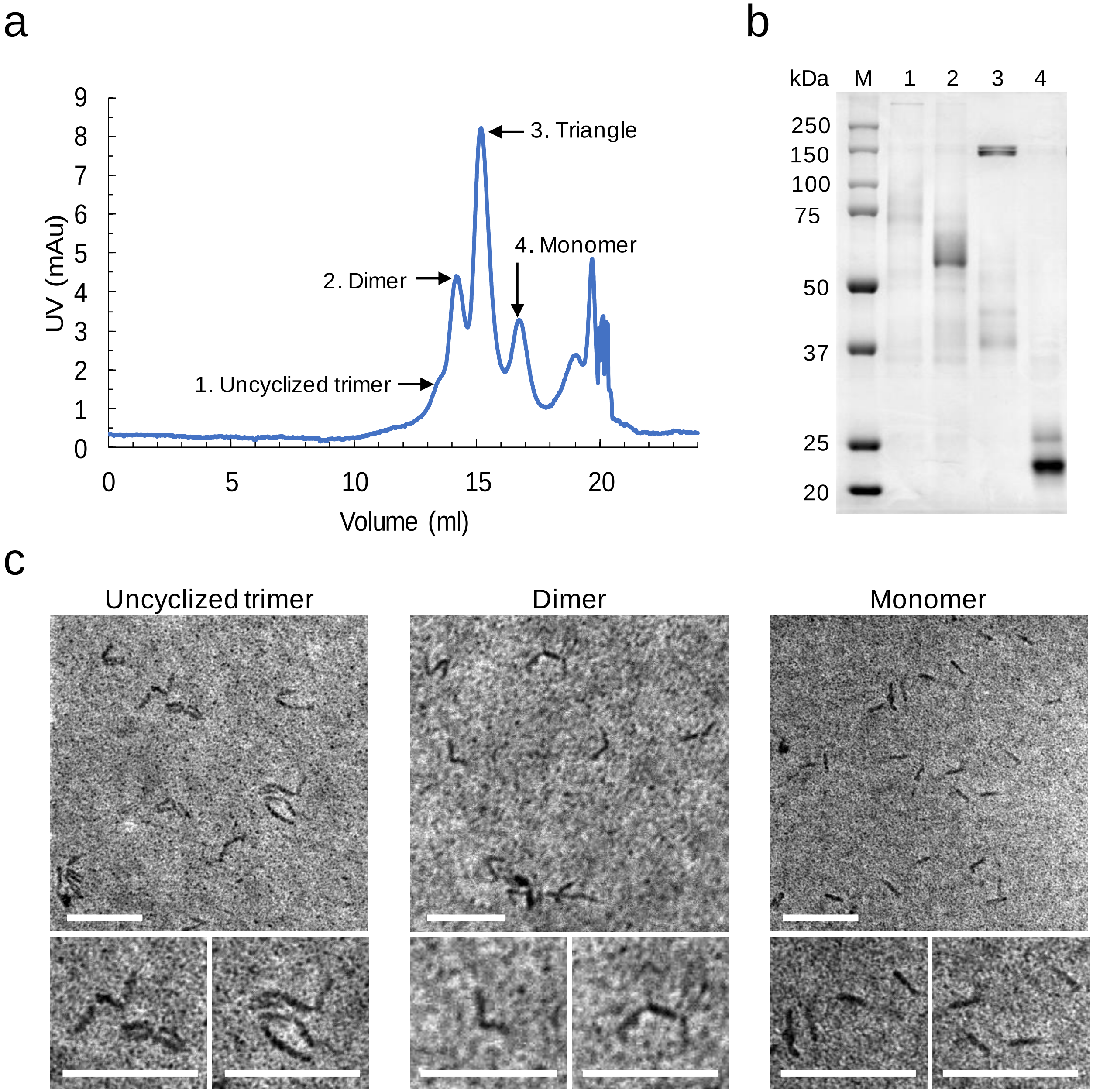

Supplementary Figure 2 Identification of side products from triangle ligation. **a** SEC profile of the triangle purification, with the peaks corresponding to protein species indicated. **b** SDS-PAGE gel showing the corresponding fractions from the SEC purification. Lane M, protein marker; lane 1, uncyclized trimer; lane 2, dimer; lane 3, triangular nanostructure; lane 4, monomer. **c** STEM images of the three SEC fractions, showing primarily uncyclized trimer, dimer, and monomer (STEM image of triangle shown in Figure 2). Scale bars, 50 nm. Source data are provided as a Source Data file.

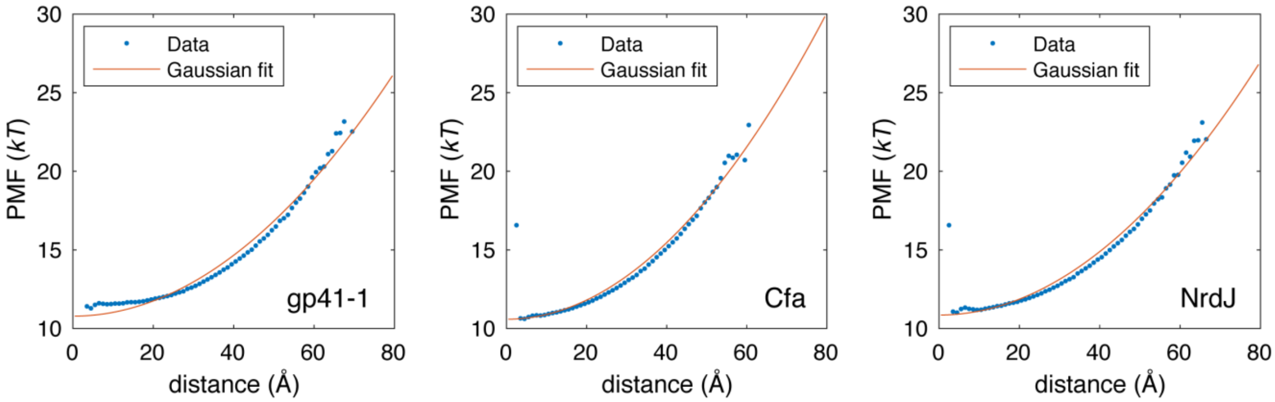

Supplementary Figure 3 Potential of mean force (PMF) data from atomistic simulations. PMF data shown as blue dots and the fitted curves as red lines. The fitting function is $f(x) = A - 3(x/R_0)^2$, where A and R_0 are fitting parameters. The Pearson correlation coefficients (R) are 0.99 (Gp), 0.97 (Cfa), and 0.97 (NrdJ). Source data are provided as a Source Data file.

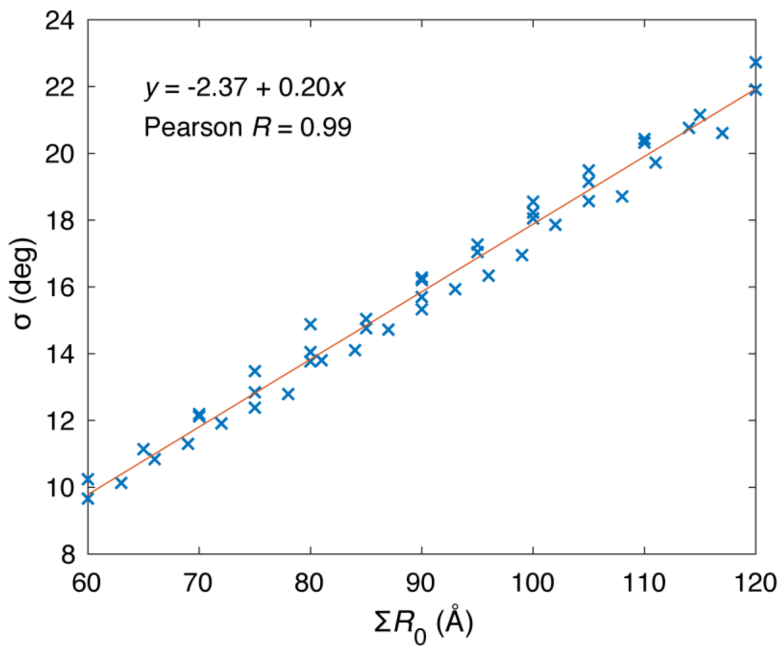

Supplementary Figure 4 Linear relationship of the summation of characteristic lengths of three Gaussian-chain linkers (ΣR_0) to the standard deviation (σ) of angles. Blue crosses indicate data points from different combinations of characteristic lengths, and the red line shows the regression line. The Pearson correlation coefficient (R) is 0.99. Source data are provided as a Source Data file.

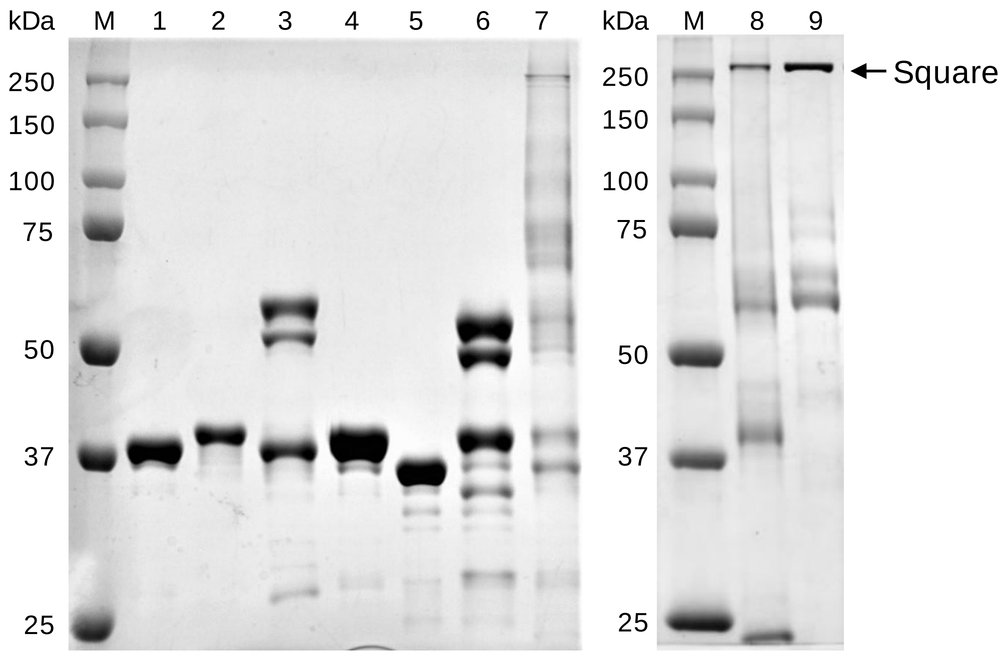

Supplementary Figure 5 SDS-PAGE gel of the construction and purification of the square nanostructure. Lane M, protein marker; lane 1, purified Gp^{C}_{10}-3HBt-Cfa^{N}_{10}; lane 2, purified Cfa^{C}_{10}-3HBt-$NrdJ^{N}_{10}$; lane 3, ligation product of Gp^{C}_{10}-3HBt-Cfa^{N}_{10} and Cfa^{C}_{10}-3HBt-$NrdJ^{N}_{10}$; lane 4, purified $NrdJ^{C}_{10}$-3HBt-Cfa^{N}_{10}; lane 5, purified Cfa^{C}_{10}-3HBt-Gp^{N}_{10}; lane 6, ligation product of $NrdJ^{C}_{10}$-3HBt-Cfa^{N}_{10} and Cfa^{C}_{10}-3HBt-Gp^{N}_{10}; lane 7, ligation product of Gp^{C}_{10}-3HBt-Cfa^{N}_{10}, Cfa^{C}_{10}-3HBt-$NrdJ^{N}_{10}$, $NrdJ^{C}_{10}$-3HBt-Cfa^{N}_{10}, and Cfa^{C}_{10}-3HBt-Gp^{N}_{10}; lane 8, square purified by Ni-NTA column; lane 9, square further purified by SEC. Source data are provided as a Source Data file.

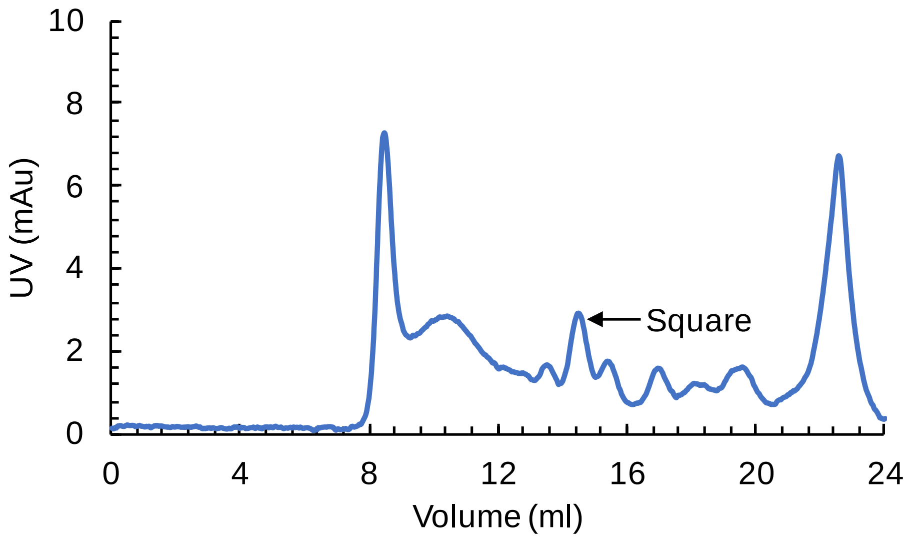

Supplementary Figure 6 SEC profile of the square purification. The peak corresponding to the desired square nanostructure is indicated. Source data are provided as a Source Data file.

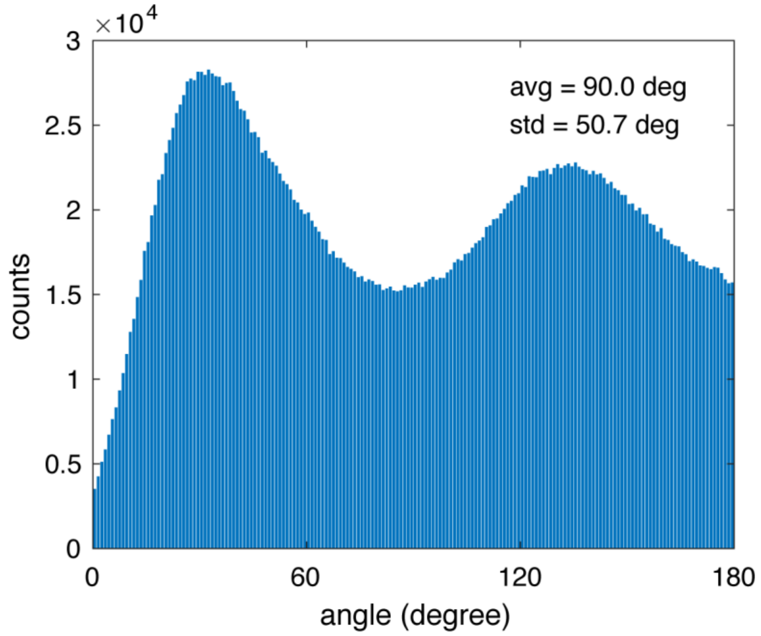

Supplementary Figure 7 Simulated distribution of the square structure angles with no constraints on angle flexibility. Note that the simulated standard deviation (50.7°) is significantly higher than the measured value (27.6°) and that the simulation produces a bimodal distribution instead of the trimodal distribution observed experimentally. Source data are provided as a Source Data file.

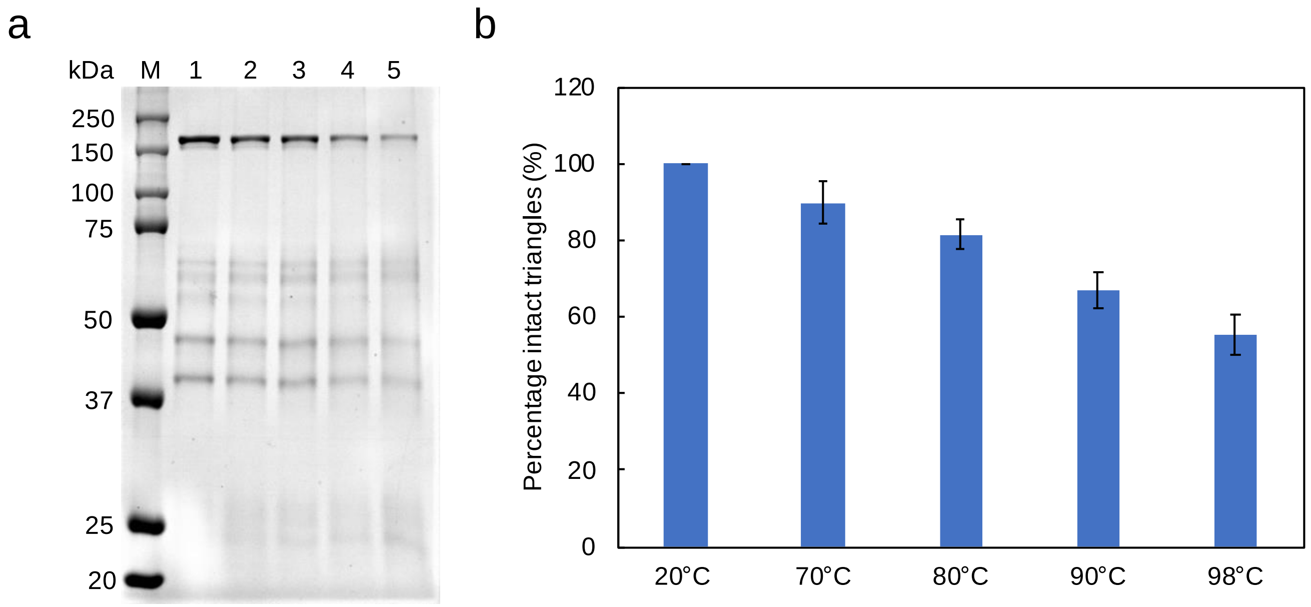

Supplementary Figure 8 SDS-PAGE analysis of protein triangle thermostability. **a** Representative SDS-PAGE gel showing Tri10t sample before and after incubation at elevated temperatures. Lane M, protein marker; lane 1, Tri10t incubated at 20°C; lane 2, Tri10t incubated at 70°C; lane 3, Tri10t incubated at 80°C; lane 4, Tri10t incubated at 90°C; lane 5, Tri10t incubated at 98°C. **b** Ratio of intact triangles after incubation at different temperatures based on band intensities observed in SDS-PAGE. Temperature melts were performed in triplicate and normalized by the sample incubated at 20°C. Error bars represent the s.d. (n = 3 temperature melt replicates). Source data are provided as a Source Data file.

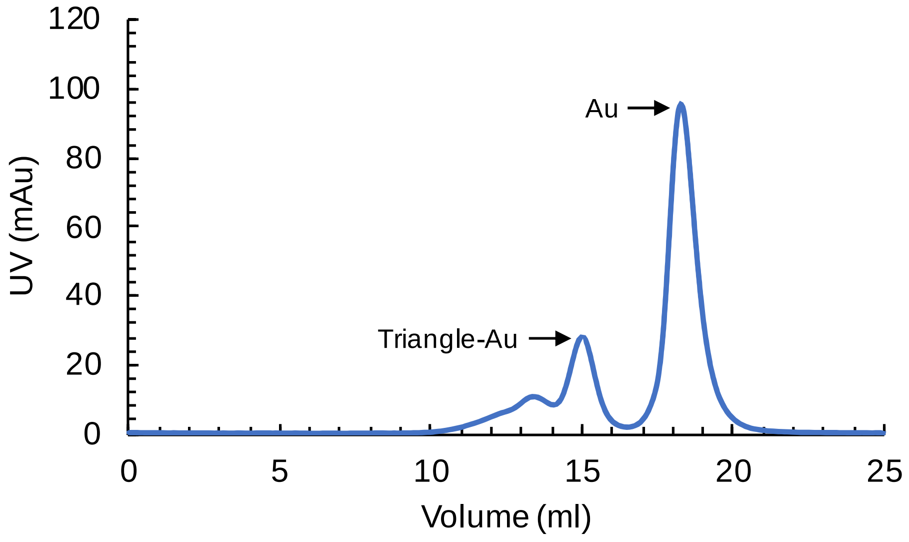

Supplementary Figure 9 SEC profile of the purification of the triangular nanostructures labeled with AuNPs. Peaks corresponding to the labeled nanostructures (Triangle-Au) and unbound AuNPs (Au) are identified. Source data are provided as a Source Data file.

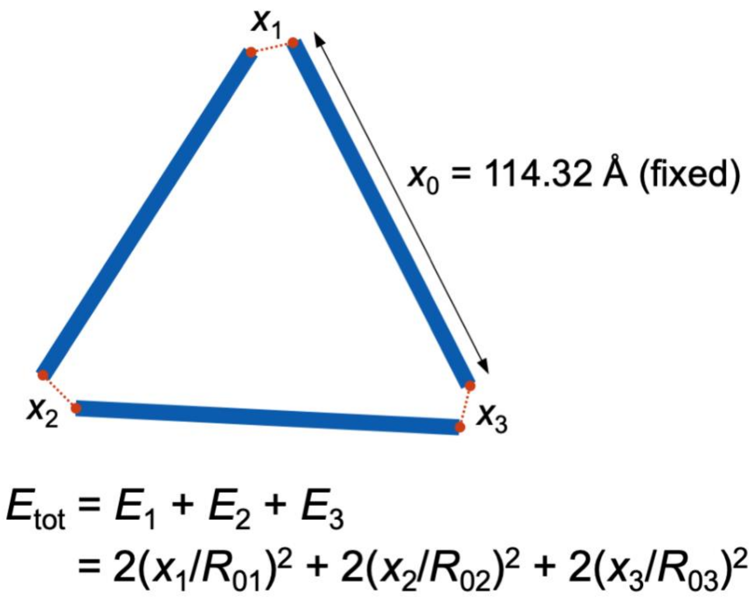

Supplementary Figure 10 Schematic representation of the triangular structures used in coarse-grained simulations. The main part of 3HB is modeled as a rigid rod with fixed length of 114.32 Å, and the flexible linker is modeled as a 2-dimensional harmonic spring. The total energy of the system is a simple arithmetic sum of all spring energies.

Supplementary Tables

Supplementary Table 1 Linker information of triangular and square nanostructures.

Protein nanostructure	Protein building block	Linker length	Linker amino acid sequence
Tri18	Gp$^{C}_{18}$-3HB-Cfa$^{N}_{14}$	14	Cfa$_{14}$: SGPGAEYCFNSGPG
Tri18	Cfa$^{C}_{14}$-3HB-NrdJ$^{N}_{18}$	18	NrdJ$_{18}$: SGPGGTNPCSEIVLSGPG
Tri18	NrdJ$^{C}_{18}$-3HB-Gp$^{N}_{18}$	18	Gp$_{18}$: SGPGTRSGYSSSDVSGPG
Tri10	Gp$^{C}_{10}$-3HB-Cfa$^{N}_{10}$	10	Cfa$_{10}$: PGAEYCFNGP
Tri10	Cfa$^{C}_{10}$-3HB-NrdJ$^{N}_{10}$	10	NrdJ$_{10}$: PGNPCSEIGP
Tri10	NrdJ$^{C}_{10}$-3HB-Gp$^{N}_{10}$	10	Gp$_{10}$: PGSGYSSSGP
Tri7	Gp$^{C}_{7}$-3HB-Cfa$^{N}_{7}$	7	Cfa$_{7}$: PGCFNGP
Tri7	Cfa$^{C}_{7}$-3HB-NrdJ$^{N}_{7}$	7	NrdJ$_{7}$: PGSEIGP
Tri7	NrdJ$^{C}_{7}$-3HB-Gp$^{N}_{7}$	7	Gp$_{7}$: PGSSSGP
Tri18t	Gp$^{C}_{18}$-3HB$_{t}$-Cfa$^{N}_{14}$	14	Cfa$_{14}$: SGPGAEYCFNSGPG
Tri18t	Cfa$^{C}_{14}$-3HB$_{t}$-NrdJ$^{N}_{18}$	18	NrdJ$_{18}$: SGPGGTNPCSEIVLSGPG
Tri18t	NrdJ$^{C}_{18}$-3HB$_{t}$-Gp$^{N}_{18}$	18	Gp$_{18}$: SGPGTRSGYSSSDVSGPG
Tri14t	Gp$^{C}_{14}$-3HB$_{t}$-Cfa$^{N}_{10}$	10	Cfa$_{10}$: PGAEYCFNGP
Tri14t	Cfa$^{C}_{10}$-3HB$_{t}$-NrdJ$^{N}_{14}$	14	NrdJ$_{14}$: PGGTNPCSEIVLGP
Tri14t	NrdJ$^{C}_{14}$-3HB$_{t}$-Gp$^{N}_{14}$	14	Gp$_{14}$: PGTRSGYSSSDVGP
Tri10t	Gp$^{C}_{10}$-3HB$_{t}$-Cfa$^{N}_{10}$	10	Cfa$_{10}$: PGAEYCFNGP
Tri10t	Cfa$^{C}_{10}$-3HB$_{t}$-NrdJ$^{N}_{10}$	10	NrdJ$_{10}$: PGNPCSEIGP
Tri10t	NrdJ$^{C}_{10}$-3HB$_{t}$-Gp$^{N}_{10}$	10	Gp$_{10}$: PGSGYSSSGP
Square	Gp$^{C}_{10}$-3HB$_{t}$-Cfa$^{N}_{10}$	10	Cfa$_{10}$: PGAEYCFNGP
Square	Cfa$^{C}_{10}$-3HB$_{t}$-NrdJ$^{N}_{10}$	10	NrdJ$_{10}$: PGNPCSEIGP
Square	NrdJ$^{C}_{10}$-3HB$_{t}$-Cfa$^{N}_{10}$	10	Cfa$_{10}$: PGAEYCFNGP
Square	Cfa$^{C}_{10}$-3HB$_{t}$-Gp$^{N}_{10}$	10	Gp$_{10}$: PGSGYSSSGP

Note: Residual extein sequences, specific to the SI group used, are underlined.

Supplementary Table 2 Primer sequences used in plasmid construction.

Primer name	Primer sequence
prWB01	GGTCTCGGTCCAGGTAATGAAGACGACATGAAAAAACTG
prWB02	GGTCTCTCCGGATTCGAGGCCTTTCAGCACTTTTTCG
prWB03	GGTCTCCATATGGCTAAGACTAAAATGCTGAAAAAAATTC
prWB04	GGTCTCTGGACCGGAAACATCGCTAGAAGAGTTGTGG
prWB05	GGTCTCTCCGGACCGGGTGCAGAATATTGCCTGTCTTACG
prWB06	GGTCTCGCTACCCGGCAAACCATCAACTTGTTTCAGG
prWB07	GGTCTCCATATGTATATCTCCTTCTTAAAAGATCTTTTGAATTC
prWB08	GGTCTCGGTAGCCATCACCATCACCATCATCACCACC
prWB09	GGTCTCCATATGGCTAAGACTAAAGTCAAGATC
prWB10	GGTCTCTGGACCGGAGTTGAAGCAGTTAGAGGCCAC
prWB11	GGTCTCTCCGGACCGGGTGGCACTAACCCGTGCTG
prWB12	GGTCTCGCTACCAATAGCCACCACCAGTTC
prWB13	GGTCTCCATATGGCTAAGACTAAAGAGGCGAAG
prWB14	GGTCTCTGGACCGGACAGCACGATTTCGGAGTTG
prWB15	GGTCTCTCCGGACCGGGTACCCGTTCTGGTTACTGTCTG
prWB16	GGTCTCGCTACCTTCTTTCACGTACAGGCACATAC
prWB17	GGTCTCGGTCCAAATGAAGACGACATGAAAAAACTG
prWB18	GGTCTCACCCGGTTCGAGGCCTTTCAGCACTTTTTC
prWB19	GGTCTCTGGACCGCTAGAAGAGTTGTGGGTCAG
prWB20	GGTCTCCCGGGTGCAGAATATTGCCTGTCTTACG
prWB21	GGTCTCTGGACCGTTGAAGCAGTTAGAGGCCAC
prWB22	GGTCTCCCGGGTAACCCGTGCTGCCTGGTTG
prWB23	GGTCTCTGGACCGATTTCGGAGTTGTGTACCAGAATG
prWB24	GGTCTCCCGGGTTCTGGTTACTGTCTGGACCTG
prWB25	GGTCTCCCGGGTTGCCTGTCTTACGACACAGAG
prWB26	GGTCTCCCGGGTTGCTGCCTGGTTGGCAGC
prWB27	GGTCTCCCGGGTTGCTGTCTGGACCTGAAGACCC
prWB28	GGTCTCGGTCCAGGTAAAAAACTGTATAAACAAATGGTGCAGG
prWB29	GGTCTCTCCGGATTTCAGCACTTTTTCGGCGATTTC
prWB30	GGTCTCGGTCCAAAAAAACTGTATAAACAAATGGTGCAG
prWB31	GGTCTCACCCGGGCCGCACAGCACTTTTTCGGCGA
prWB32	GGTCTCTGGACCAACATCGCTAGAAGAGTTGTG
prWB33	GGTCTCCCGGGTGGCACTAACCCGTGCTGC
prWB34	GGTCTCTGGACCCAGCACGATTTCGGAGTTG
prWB35	GGTCTCCCGGGTACCCGTTCTGGTTACTGTCTGGAC

Supplementary Table 3 Percentages of component structures present following purification by affinity chromatography and SEC, as determined by SDS-PAGE gel densitometry.

Component	After affinity chromatography (%)	After SEC (%)
Triangle or square	60.4	81.0
Uncyclized trimer	0.6	ND
Dimer	21.0	8.3
Monomer with SI	10.0	10.7
Monomer without SI	8.1	ND

Note: ND, not detected. The relative amounts of the components of triangular nanostructure sample were quantified based on Supplementary Figure 1. Source data are provided as a Source Data file.

Supplementary Table 4 Secondary structure contents of simulated linker proteins, as quantified by the DSSP scores.

Name	Sequence	Avg. α content (H-score)	Avg. β content (E-score)
$3HB^{C}$-Cfa_{14}-$3HB^{N}$	*GLE*SGPGAEYCFNSGPG**NEDDM**	8.04 %	1.42 %
$3HB^{C}$-$NrdJ_{18}$-$3HB^{N}$	*GLE*SGPGGTNPCSEIVLSGPG**NEDDM**	3.47 %	1.95 %
$3HB^{C}$-Gp_{18}-$3HB^{N}$	*GLE*SGPGTRSGYSSSDVSGPG**NEDDM**	2.05 %	3.06 %

Note: The flexible N- and C-terminal tail regions of 3HB are shown in bold and italic text, respectively.

Supplementary Table 5 Gaussian characteristic lengths of linkers extracted from atomistic simulations. The fitting function is $f(x) = A - 3(x/R_0)^2$, where A and R_0 are fitting parameters.

Name	Sequence	R_0 (Å)	Pearson's R
Cfa_{10}	PGAEYCFNGP	20.3	0.86
$NrdJ_{10}$	PGNPCSEIGP	20.1	0.88
Gp_{10}	PGSGYSSSGP	21.7	0.83
$NrdJ_{14}$	PGGTNPCSEIVLGP	24.6	0.97
Gp_{14}	PGTRSGYSSSDVGP	24.3	0.93
Cfa_{14}	SGPGAEYCFNSGPG	23.1	0.96
$NrdJ_{18}$	SGPGGTNPCSEIVLSGPG	28.8	0.96
Gp_{18}	SGPGTRSGYSSSDVSGPG	27.7	0.97
$3HB^C$-Cfa_{10}-$3HB^N$	*GLE*PGAEYCFNGP**NEDDM**	28.0	1.00
$3HB^C$-$NrdJ_{10}$-$3HB^N$	*GLE*PGNPCSEIGP**NEDDM**	29.0	0.97
$3HB^C$-Gp_{10}-$3HB^N$	*GLE*PGSGYSSSGP**NEDDM**	30.4	0.95
$3HB^C$-Cfa_{14}-$3HB^N$	*GLE*SGPGAEYCFNSGPG**NEDDM**	31.4	0.97
$3HB^C$-$NrdJ_{18}$-$3HB^N$	*GLE*SGPGGTNPCSEIVLSGPG**NEDDM**	35.2	0.99
$3HB^C$-Gp_{18}-$3HB^N$	*GLE*SGPGTRSGYSSSDVSGPG**NEDDM**	34.5	0.97

Note: The flexible N- and C-terminal tail regions of 3HB are shown in bold and italic text, respectively.
