# Supplementary material for: Covalently-assembled single-chain protein nanostructures with ultra-high stability
Source: Nat Commun. 2019 Jul 25;10:3317. doi: 10.1038/s41467-019-11285-8 (PMC6658521; doi:10.1038/s41467-019-11285-8)
Supplement: Supplementary file 3 — Description of Additional Supplementary Files [file 41467_2019_11285_MOESM3_ESM.pdf]

Description of Additional Supplementary Files

File name: Supplementary Data 1

Description: Primers and plasmids used in expression plasmid construction.

File name: Supplementary Data 2

Description: Amino acid sequences of protein building blocks used to construct the triangular and square nanostructures.
